# Higher dietary protein intake is associated with sarcopenia in older British twins

**DOI:** 10.1093/ageing/afad018

**Published:** 2023-02-14

**Authors:** Mary Ni Lochlainn, Ruth C E Bowyer, Ailsa A Welch, Kevin Whelan, Claire J Steves

**Affiliations:** Department of Twin Research and Genetic Epidemiology, King’s College London, London SE1 7EH, UK; Department of Twin Research and Genetic Epidemiology, King’s College London, London SE1 7EH, UK; AI for Science and Government, The Alan Turing Institute, London NW1 2DB, UK; Department of Public Health and Primary Care, Norwich Medical School, University of East Anglia, Norwich NR4 7TJ, UK; Department of Nutritional Sciences, King’s College London, London SE1 9NH, UK; Department of Twin Research and Genetic Epidemiology, King’s College London, London SE1 7EH, UK

**Keywords:** sarcopenia, frailty, protein, gut microbiome, muscle loss, older people

## Abstract

**Background:**

Sarcopenia, characterised by an accelerated loss of skeletal muscle mass and function, is associated with negative outcomes. This study aimed to evaluate factors associated with skeletal muscle strength, mass and sarcopenia, particularly protein intake, and to assess whether shared twin characteristics are important.

**Methods:**

This study utilised cross-sectional data from a study of community-dwelling twins aged ≥60 years. Multivariable logistic regression and between- and within-twin pair regression modelling were used.

**Results:**

Participants (*n* = 3,302) were 89% female (*n* = 2,923), aged a mean of 72.1 (±7.3) years and composed of 858 (55%) monozygotic, 709 (45%) dizygotic twin pairs and 168 individual lone twins. Using optimal protein intake as the reference group (1.0–1.3 g/kg/day), there was no significant association between protein intake (neither high nor low) and low muscle strength, or between low protein intake and sarcopenia (odds ratio (OR) 0.7; 95% confidence interval (CI) 0.39–1.25; *P* = 0.229) in unadjusted models. High protein intake (>1.3 g/kg/day) was associated with low muscle mass (OR 1.76; 95% CI 1.39–2.24; *P* < 0.0001), while low protein intake was protective (OR 0.52; 95% CI 0.40–0.67; *P* < 0.0001). High protein intake was associated with sarcopenia (OR 2.04; 95% CI 1.21–3.44; *P* = 0.008), and this was robust to adjustment for demographic, anthropometric and dietary factors. The association between muscle strength and weight, body mass index, healthy eating index, protein intake and alpha diversity was not significantly influenced by shared twin factors, indicating greater amenability to interventions.

**Conclusions:**

High protein intake is associated with sarcopenia in a cohort of healthy older twins.

## Key Points

- High protein intake is associated with sarcopenia, even after adjustment for a range of covariates.
- High protein intake was associated with low muscle mass, while a low intake was protective.
- Among other factors, muscle strength is associated with age, education, income, diet, appetite and gut microbiota diversity.
- Associations with body mass index, diet and microbiome diversity are not significantly explained by twin factors, therefore more modifiable.
- We report a sarcopenia prevalence of 4.3% in a cohort of community-dwelling volunteer twins, aged 60 and older.

## Introduction

Muscle loss with age is a growing problem, particularly as populations around the world are aging. Sarcopenia refers to a progressive and generalised skeletal muscle disorder involving the accelerated loss of skeletal muscle function and is associated with increased adverse outcomes including falls, functional decline, frailty, reduced quality of life, higher healthcare costs and mortality [1, 2]. Data on its prevalence vary widely, ranging from 1 to 31.9% [3–5].

Despite the significant burden of sarcopenia, there are limited therapeutic options available. Much of the literature investigates resistance exercise and protein supplementation as the main treatment approaches, with compelling evidence for resistance exercise and less consistent evidence for protein [6]. Beyond resistance exercise and protein intake, many features have been associated with sarcopenia, including smoking [7], education [8], income [8], sex [9], diet [1], appetite [10], frailty [1] and physical activity [1].

Anabolic resistance refers to the phenomenon whereby older adults require a higher dose of protein to achieve the same response in muscle protein synthesis as younger adults [11]. This has led to the European Society for Clinical Nutrition and Metabolism (ESPEN) producing guidance recommending a higher daily intake of protein (1–1.3 g/kg/day) for older adults, in order to overcome this resistance [12, 13].

The gut microbiota and their role in human physiology are a growing field of enquiry, with microbiota diversity typically considered as a marker of overall health. There is an expanding body of evidence linking the gut microbiota to skeletal muscle function, which we have described in full previously [14]. The gut microbiota play a key role in many of the postulated mechanisms and aetiologies for anabolic resistance, for example, gut permeability, and inflammation, leading to the suggestion that the microbiota may mediate anabolic resistance to some degree [14]. To our knowledge, there have been no previous studies that have investigated the association between the broad range of characteristics investigated in our study, such as healthy eating index, frailty, appetite, indicators of renal function, gut microbiota diversity, and the relevance of shared twin factors with sarcopenia. Therefore, the goals of this study, established a priori, were to (i) ascertain the prevalence of low muscle strength and sarcopenia in a large cohort of British twins aged ≥60 years; (ii) explore factors associated with low muscle strength and sarcopenia, in particular, dietary protein intake and (iii) use specialised regression methods to explore whether shared twin factors (e.g. genetics, early environment, etc.) drive the identified associations with muscle strength and/or sarcopenia. While aims (i) and (ii) have been explored in other populations to some degree, aim (iii) has not been done before to our knowledge, and this study represents the first piece of research using twin modelling in this field of enquiry.

## Methods

### Study population

The current study utilises a cross-sectional sample of community-dwelling participants in the TwinsUK cohort who had detailed data available on skeletal muscle mass, muscle strength, physical performance, diet and anthropometry (*n* = 3,302). The TwinsUK cohort has been described in detail elsewhere [15]. Eligibility for the analysis was defined by being aged ≥60 years and an attendance for a visit to the department since 2010 which included detailed physical measures, DXA scans and questionnaire completion. There were no exclusion criteria.

A logistic regression approach was used for the main analyses. For the twin modelling analysis, linear regression was used. A Wald test was used to test the difference between the between-pair and within-pair coefficients. Variable measurement and statistical analysis are described in full in the appendices.

## Results

**Figure 1:**
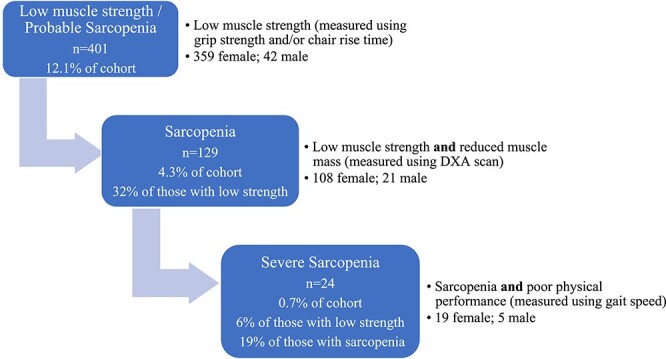
Prevalence of sarcopenia [1].

A total of 3,302 individual twins were included, with a mean age of 72.1. The overall prevalence of sarcopenia in this cohort was 129 (4.3%), including 21 (6.2%) males and 108 (4.1%) females (Figure 1) [1, 16].

### Factors associated with muscle strength and sarcopenia

**Figure 2:**
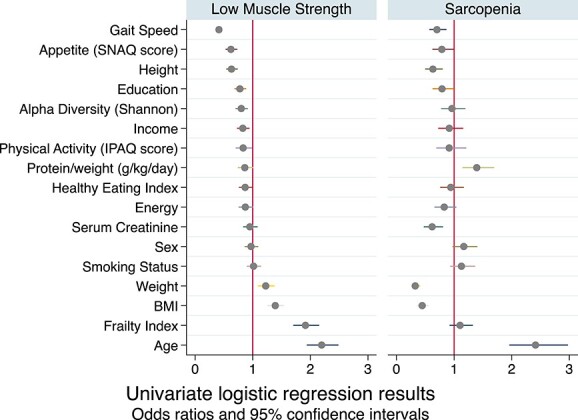
Logistic regression results for covariates of low muscle strength and sarcopenia. All models adjusted for age and sex. All variables were standardised therefore each unit of difference refers to one standard deviation of difference for that variable. The prevalence of low muscle strength is 12.1%. The prevalence of sarcopenia is low (4.3% of this cohort) which will impact power in these analyses.

When comparing those with low muscle strength to those without, there was no difference between the two groups in protein intake, using both UK Reference Nutrient Intake (RNI) and ESPEN recommended intakes (Table 1). Body mass index (BMI) was significantly lower in the participants with sarcopenia compared to those without sarcopenia. In terms of protein intake, both measures of protein intake (UK RNI and ESPEN) were significantly different, with those with sarcopenia more likely to have ‘high’ protein intake. Figure 2 presents the logistic regression analysis for the relationship between each variable and muscle strength, defined as low or not, and sarcopenia (see also Supplementary Table 3).

**Table 1:** Population characteristics and factors associated with low muscle strength, low muscle mass and sarcopenia

	Total (*n* = 3,302)	Normal muscle strength (*n* = 2,901, 87.9%)	Low muscle strength (*n* = 401, 12.1%)	*P*-value	Normal muscle mass (*n* = 2024,	Low muscle mass (*n* = 967,	*P*-value	No Sarcopenia (*n* = 2,862, 95.7%)	Sarcopenia (*n* = 129, 4.3%)	*P*-value
Age (years), mean (SD)	72.1 (7.3)	71.4 (6.9)	77.3 (8.3)	*<0.001*	71.3 (7.0)	73.0 (7.3)	*<0.001*	71.6 (6.9)	78.2 (8.3)	*<0.001*
Sex, *n* (%)				0.5			*<0.001*			0.066
Female	2,923 (89%)	2,564 (88%)	359 (90%)		1,754 (87%)	900 (93%)		2,546 (89%)	108 (84%)	
Male	379 (11%)	337 (12%)	42 (10%)		270 (13%)	67 (7%)		316 (11%)	21 (16%)	
Zygosity, *n* (%)				0.59			0.56			0.31
Monozygotic	1,787 (54%)	1,565 (54%)	222 (55%)		1,097 (54%)	535 (55%)		1,556 (54%)	76 (59%)	
Dizygotic	1,515 (46%)	1,336 (46%)	179 (45%)		927 (46%)	432 (45%)		1,306 (46%)	53 (41%)	
Highest education level achieved, *n* (%)				*<0.001*			*0.005*			*<0.001*
Low	1,423 (48%)	1,194 (45%)	229 (63%)		822 (45%)	455 (51%)		1,200 (46%)	77 (64%)	
Middle	921 (31%)	830 (32%)	91 (25%)		597 (33%)	245 (28%)		815 (31%)	27 (22%)	
High	651 (22%)	605 (23%)	46 (13%)		406 (22%)	185 (21%)		574 (22%)	17 (14%)	
Annual household income, *n* (%)				*<0.001*			*0.022*			0.081
Declined to answer	607 (21%)	512 (20%)	95 (26%)		312 (20%)	192 (25%)		522 (21%)	29 (26%)	
Low	1,106 (39%)	928 (37%)	178 (49%)		608 (39%)	309 (40%)		947 (38%)	51 (45%)	
Middle	696 (24%)	637 (25%)	59 (16%)		398 (25%)	168 (21%)		628 (25%)	20 (18%)	
High	451 (16%)	423 (17%)	28 (8%)		253 (16%)	113 (14%)		412 (16%)	13 (12%)	
Smoking status, *n* (%)				0.69			0.11			0.49
Never smoked	1,886 (58%)	1,649 (57%)	237 (60%)		1,095 (57%)	566 (61%)		1,644 (58%)	68 (53%)	
Ex-smoker	1,215 (37%)	1,075 (37%)	140 (35%)		745 (39%)	323 (35%)		1,046 (37%)	54 (42%)	
Current smoker	165 (5%)	145 (5%)	20 (5%)		87 (5%)	43 (5%)		139 (5%)	6 (5%)	
Weight (kg), mean (SD)	70 (14)	70 (14)	71 (15)	0.23	74.5 (13.4)	60.2 (7.9)	*<0.001*	70 (14)	61 (8)	*<0.001*
Height (m), mean (SD)	1.63 (0.08)	1.63 (0.07)	1.60 (0.08)	*<0.001*	1.63 (0.08)	1.62 (0.07)	*0.009*	1.63 (0.07)	1.60 (0.08)	*<0.001*
BMI (kg/m^2^), mean (SD)	27 (5)	26 (5)	28 (5)	*<0.001*	28.1 (4.8)	22.9 (2.6)	*<0.001*	27 (5)	24 (3)	*<0.001*
Serum creatinine (μmol/l), mean (SD)	74.4 (15.5)	74.3 (14.8)	75.5 (20.3)	0.14	76.0 (15.8)	70.9 (13.7)	<0.001	74.5 (15.2)	72.1 (18.1)	0.094
Creatinine Clearance (ml/min), mean (SD)	68.7 (19.2)	69.2 (18.4)	65.2 (23.7)	*<0.001*	72.7 (19.5)	60.7 (14.7)	*<0.001*	69.3 (18.8)	58.4 (19.7)	*<0.001*
Healthy eating index, mean (SD)	61 (10)	61 (10)	59 (9)	*0.002*	60.7 (9.1)	61.4 (10.0)	0.18	61 (10)	59 (10)	0.069
Protein intake adequacy (RNI), *n* (%)				0.27			<0.001			*0.009*
Optimal (≥0.75 g/kg/day)	1,720 (87%)	1,526 (88%)	194 (85%)		1,041 (84.5%)	595 (93.4%)		1,552 (87%)	84 (97%)	
Low (<0.75 g/kg/day)	249 (13%)	215 (12%)	34 (15%)		191 (15.5%)	42 (6.6%)		320 (13%)	3 (3%)	
Protein Intake (ESPEN), *n* (%)				0.60			<0.001			*<0.001*
Low (<1 g/kg/day)	733 (37%)	644 (37%)	89 (39%)		386 (31%)	204 (32%)		671 (38%)	19 (22%)	
Optimal (1–1.3 g/kg/day)	619 (31%)	554 (32%)	65 (29%)		541 (44%)	149 (23%)		567 (32%)	23 (26%)	
High (>1.3 g/kg/day)	617 (31%)	543 (31%)	74 (32%)		305 (25%)	284 (45%)		544 (31%)	45 (52%)	
Energy intake (kcal/day), mean (SD)	1757.7 (445.9)	1761.8 (449.7)	1726.5 (415.9)	0.26	1770.0 (447.2)	1743.9 (450.4)	0.23	1762.7 (449.3)	1727.9 (429.2)	0.48
Muscle mass (appendicular lean mass/height^2^), mean (SD)	6.1 (1.0)	6.1 (1.0)	6.0 (0.9)	0.15	6.5 (0.9)	5.2 (0.5)	*<0.001*	6.1 (1.0)	5.3 (0.6)	*<0.001*
Gait speed (m/s), mean (SD)	1.1 (0.2)	1.2 (0.2)	0.9 (0.3)	*<0.001*	1.1 (0.2)	1.1 (0.2)	0.49	1.1 (0.2)	1.0 (0.3)	*<0.001*
Physical activity (IPAQ), mean (SD)	2.1 (0.8)	2.1 (0.8)	1.9 (0.8)	*0.002*	2.1 (0.8)	2.1 (0.8)	0.5	2.1 (0.8)	2.0 (0.8)	0.21
Frailty Index, mean (SD)	0.2 (0.1)	0.2 (0.1)	0.3 (0.2)	*<0.001*	0.2 (0.1)	0.2 (0.1)	*0.025*	0.2 (0.1)	0.3 (0.1)	*<0.001*
Appetite (SNAQ), mean (SD)	15.4 (1.8)	15.5 (1.7)	14.4 (2.4)	*<0.001*	15.5 (1.7)	15.2 (1.8)	* 0.003*	15.4 (1.7)	14.9 (1.9)	*0.017*
Alpha diversity (Shannon), mean (SD)	5.2 (0.7)	5.2 (0.7)	5.1 (0.7)	*0.007*	5.16 (0.72)	5.23 (0.70)	* 0.046*	5.2 (0.7)	5.2 (0.7)	0.87

*Italics* = statistically significant; RNI, Reference Nutrient Intake; ESPEN, European Society for Clinical Nutrition and Metabolism; IPAQ, International Physical Activity Questionnaire; SNAQ, Simplified Nutritional Appetite Questionnaire.

### Sarcopenia, but not muscle strength, is associated with protein intake

The results of multivariable logistic regression analyses used to determine adjusted odds ratios (ORs) for the relation between protein intake and low muscle strength, low muscle mass and sarcopenia are presented in Table 2. There was no significant association between protein intake and muscle strength in any of the models. There was a significant association between protein intake (high and low, when compared to the reference category) and muscle mass, robust to adjustment in all models. Low protein intake was* protective* of low muscle mass (OR 0.52; 95% confidence interval (CI) 0.40–0.67; *P* < 0.0001), while high protein intake was associated with an increased odds of having low muscle mass (OR 1.76; 95% CI 1.39–2.24; *P* < 0.0001). In terms of sarcopenia, no significant association was noted for low protein intake; however, high protein intake was significantly associated with sarcopenia, and this was robust to adjustment in all models (OR 2.04; 95% CI 1.21–3.44; *P* = 0.008).

**Table 2:** ORs and 95% CIs for low muscle strength, low muscle mass and sarcopenia according to protein intake comparing low and high protein intakes to optimal protein intakes (reference)

	Low muscle strength		Low muscle mass		Sarcopenia	
Protein Intake (g/kg/day)	Low (<1 g/kg/day)	High (>1.3 g/kg/day)	Low (<1 g/kg/day)	High (>1.3 g/kg/day)	Low (<1 g/kg/day)	High (>1.3 g/kg/day)
Unadjusted	1.18 (0.84–1.65) *P* = 0.340	1.16 (0.81–1.66) *P* = 0.414	0.52 (0.40–0.67) *P < 0.0001*	1.76 (1.39–2.24) *P < 0.0001*	0.70 (0.39–1.25) *P* = 0.229	2.04 (1.21–3.44) *P = 0.008*
Model 1 *Age, sex*	1.31 (0.92–1.86) *P* = 0.131	1.10 (0.75–1.60) *P* = 0.640	0.54 (0.42–0.70) *P < 0.0001*	1.72 (1.35–2.19) *P < 0.0001*	0.77 (0.42–1.42) *P* = 0.401	2.21 (1.26–3.87) *P = 0.006*
Model 2 *1 + smoking, income, education*	1.20 (0.82–1.76) *P* = 0.349	1.02 (0.68–1.53) *P* = 0.921	0.53 (0.40–0.70) *P < 0.0001*	1.80 (1.39–2.34) *P < 0.0001*	0.75 (0.37–1.53) *P* = 0.434	2.48 (1.36–4.52) *P = 0.003*
Model 3 *2 + height*	1.23 (0.84–1.81) *P* = 0.281	0.92 (0.61–1.40) *P* = 0.702	0.53 (0.40–0.70) *P < 0.0001*	1.86 (1.43–2.41) *P < 0.0001*	0.78 (0.39–1.57) *P* = 0.489	2.36 (1.28–4.36) *P = 0.006*
Model 4: Frailty/activity *2 + frailty index + activity level (IPAQ)*	0.98 (0.60–1.58) *P* = 0.921	0.99 (0.57–1.71) *P* = 0.967	0.55 (0.38–0.79) *P = 0.001*	1.71 (1.20–2.43) *P = 0.003*	0.73 (0.28–1.92) *P* = 0.528	2.46 (1.08–5.60) *P = 0.031*
Model 5: muscle *4 + lean mass/height2*	0.92 (0.55–1.55) *P* = 0.767	1.04 (0.59–1.82) *P* = 0.889	Not done (outcome)		Not done (part of sarcopenia definition)	
Model 6: renal function *4 + creatinine clearance*	0.92 (0.57–1.51) *P* = 0.750	1.13 (0.65–1.97) *P* = 0.662	0.56 (0.39–0.81) *P = 0.002*	1.37 (0.95–1.97) *P* = 0.096	0.76 (0.30–1.95) *P* = 0.569	2.46 (1.07–5.69) *P = 0.034*
Model 7: diet *2 + energy intake (kcal/day), healthy eating index*	1.09 (0.71–1.68) *P* = 0.689	1.33 (0.71–1.77) *P* = 0.639	0.30 (0.22–0.41) *P < 0.0001*	3.01 (2.23–4.06) *P < 0.0001*	0.40 (0.19–0.86) *P = 0.019*	4.58 (2.37–8.87) *P < 0.001*
Model 8: diet *7 + SNAQ score*	1.28 (0.69–2.39) *P* = 0.433	1.70 (0.91–3.15) *P* = 0.095	0.31 (0.21–0.44) *P < 0.0001*	3.16 (2.17–4.59) *P < 0.0001*	0.49 (0.17–1.40) *P* = 0.182	5.97 (2.26–15.81) *P < 0.0001*
Model 9: Muscle and gut microbiota 4 + Shannon diversity	1.38 (0.86–2.22) *P* = 0.188	1.39 (0.84–2.28) *P* = 0.199	0.56 (0.37–0.86) *P = 0.008*	1.56 (1.04–2.35) *P = 0.033*	1.25 (0.50–3.15) *P* = 0.628	2.67 (1.20–5.95) *P = 0.016*

*Italics* = statistically significant; IPAQ, International Physical Activity Questionnaire; SNAQ, Simplified Nutritional Appetite Questionnaire.

To examine whether the results were consistent when protein was expressed as a proportion of total lean mass (g/kg FFM/day) instead of as a proportion of body weight (g/kg/day), the models were repeated for both low muscle strength and sarcopenia, with no notable differences found (Supplementary Table 4). The missingness of data is shown in Supplementary Table 5. To ascertain whether the missingness of data had any effect on this result, an analysis was carried out to assess whether variables of interest predicted the missingness of protein intake (Supplementary Table 6). Only sex predicted the missingness of the protein intake variable. As protein supplementation may have influenced our results, we noted those taking supplements. Four individuals reported taking protein supplements. Two of the four reported a low protein intake from diet, one reported a high intake, and the final one had missing data for protein intake. No subcategory analysis of this group was undertaken due to very low numbers.

### Twin modelling

For income, education, frailty and gait speed, the between-pair coefficients were larger, significantly different from zero and significantly different from the within-pair coefficients (Wald test *P* ≤ 0.05), supporting the inference that the association of these variables with muscle strength (chair-rise time) was confounded by factors that are shared by twins, such as common genes, and early life factors (Table 3).

**Table 3:** Univariable linear regression results for muscle strength: between and within models

Variable	Coefficient; 95% CI	*P* value	Wald test coefficient *P* value
Income	−0.62; [−0.81, −0.43]	*P* < 0.001	
income_between	−0.73; [−0.97, −0.50]	*P* < 0.001	−0.41 *P* = 0.036
income_within	−0.33; [−0.61, −0.04]	*P* = 0.025	
Education	−0.58; [−0.81, −0.36]	*P* < 0.001	
education_between	−0.69; [−0.96, −0.42]	*P* < 0.001	−0.52 *P* = 0.020
education_within	−0.17; [−0.53, 0.18]	*P* = 0.345	
Weight	0.06; [0.04, 0.73]	*P* < 0.001	
weight_between	0.05; [0.04, 0.07]	*P* < 0.001	−0.02 *P* = 0.242
weight_within	0.07; [0.04, 0.10]	*P* < 0.001	
BMI	0.16; [0.12, 0.21]	*P* < 0.001	
bmi_between	0.16; [0.12, 0.21]	*P* < 0.001	−0.004 *P* = 0.928
bmi_within	0.17; [0.09, 0.24]	*P* < 0.001	
Frailty index	10.89; [9.14, 12.64]	*P* < 0.001	
frailty_between	11.97; [10.13, 13.81]	*P* < 0.001	4.23 *P* = 0.008
frailty_within	7.74; [4.64, 10.84]	*P* < 0.001	
Gait speed	−8.27; [−9.35, −7.18]	*P* < 0.001	
gaitspeed_between	−8.72; [−9.80, −7.64]	*P* < 0.001	−1.64 *P* = 0.050
gaitspeed_within	−7.07; [−8.87, −5.28]	*P* < 0.001	
Physical activity (IPAQ)	−0.55; [−0.84, −0.26]	*P* < 0.001	
ipaq_between	−0.67; [−1.07, −0.28]	*P* = 0.001	−0.40 *P* = 0.116
ipaq_within	−0.28; [−0.60, 0.04]	*P* = 0.090	
Healthy eating index	−0.31; [−0.05, −0.01]	*P* = 0.006	
hei_between	−0.04; [−0.07, −0.01]	*P* = 0.009	−0.03 *P* = 0.229
hei_within	−0.01; [−0.04, 0.03]	*P* = 0.679	
Protein intake (g/kg)	−1.46; [−2.26, −0.67]	*P* < 0.001	
protein_between	−1.38; [−2.31, −0.44]	*P* = 0.004	0.36 *P* = 0.557
protein_within	−1.74; [−2.71, −0.76]	*P* < 0.001	
Appetite (SNAQ)	−0.19; [− 0.33, −0.10]	*P* = 0.006	
snaq_between	−0.72; [−1.55, 0.10]	*P* = 0.087	−0.59 *P* = 0.210
snaq_within	−0.13; [−0.31, 0.04]	*P* = 0.129	
Alpha diversity	−0.46; [−0.81, −0.12]	*P* = 0.011	
shannon_between	−0.41; [−0.89, 0.08]	*P* = 0.104	0.18 *P* = 0.630
shannon_within	−0.58; [−1.05, −0.11]	*P* = 0.016	

IPAQ, International Physical Activity Questionnaire; SNAQ, Simplified Nutritional Appetite Questionnaire.

For weight, BMI, healthy eating index, protein intake and alpha diversity, there was minimal difference between the within- and between-pair coefficients, suggesting that shared twin factors were not driving any association between these variables and muscle strength.

## Discussion

Older twins with low muscle strength had no difference in protein intake versus those without low muscle strength. In contrast, twins with sarcopenia had significantly higher protein intake than those without. High protein intake (>1.3 g/kg/day) was associated with sarcopenia, even after adjustment for a range of relevant potentially confounding variables including biological, socioeconomic and environmental exposures, muscle, and diet-related variables. These analyses were carried out using protein intake as a proportion of total body weight and were consistent when protein was considered as a proportion of total lean mass.

Considering the definition of sarcopenia is the combination of low skeletal muscle strength and reduced muscle mass, one might expect that the driving force of the association between high protein intake and sarcopenia is the association between protein intake and muscle mass. Indeed, we found protein intake (high and low, versus optimal as the reference category) was associated with low muscle mass (as defined by the EWGSOP2 cutoffs for men and women), and this was robust to adjustment in all models. High protein intake was associated with increased odds of low muscle mass, and low protein intake was associated with reduced odds (i.e. protective) of having low muscle mass. However, for sarcopenia, the association was only found for ‘high’ protein intake. This suggests that the established relationship between protein intake and muscle mass does not explain all of the relationship seen, and there is a unique relationship between the sarcopenic phenotype, the combination of reduced mass and strength, that is associated with an excessive dietary protein intake, which warrants further exploration.

A recent longitudinal study also reported a negative effect of high protein intake, with higher protein intake from animal sources associated with a deterioration in health-related quality of life scores over 12 years [17]. In terms of muscle strength in particular, data from the Hertfordshire Cohort Study found that higher grip strength was associated with ‘lower’ meat consumption in men, while those with diets characterised by high consumption of fruit, vegetables, and fatty fish had higher grip strength in both men and women [18]. Similarly, in the Newcastle 85+ Study, dietary patterns high in characteristic British foods, including red meat, and with protein intake >1 g/kg/day were associated with an increased risk of sarcopenia [19]. Most of the available literature focuses on inadequate protein intake [14], as this is more common. Many studies treat protein intake as a binary variable, either below or meeting the RNI, and thus do not consider those with high intakes. It is plausible that this association is due to those individuals with sarcopenia deliberately consuming more protein to ameliorate their muscle loss. Considering sarcopenia is not routinely diagnosed in clinical practice [20], one might consider this unlikely; however, these individuals may have had another event that led to a dietician referral, so it cannot be ruled out. It is worth highlighting that our cohort has a healthy volunteer bias, with a healthier diet and higher protein intake than average, and therefore is distinct from a clinical inpatient or multi-morbid and/or frail population. Thus, our results indicate that for older adults who are relatively ‘healthy’, exceeding recommended protein intake may possibly be more detrimental to muscle health than insufficient protein intake.

While not specific to older adults, there is existing evidence of detrimental effects of high protein intake, including coronary artery disease, cancer, disorders of liver and renal function, and disorders of bone and calcium metabolism [21]. Furthermore, a growing body of evidence has emerged, linking energy restriction to longevity and healthy aging, as well as a reduced risk of diseases including type 2 diabetes and ischemic heart disease [22]. There is evidence that diets with restricted protein and/or specific amino acids are associated with improved health span and that protein may be the driving factor behind the benefits of energy restriction, via its effects on the IGF-1/mTOR network [23]. This should also be considered in clinical recommendations on protein intake.

Not all sources of protein contain the full range of essential amino acids, and the quantity of leucine varies by protein source [24]. The environmental impact of animal sources of protein, particularly red meat, in the context of the global climate crisis must also be considered. There is an ongoing debate about the ideal protein source for older adults, and a recent review suggests a mix of sources is likely to be the best approach [25]. Using the same method used to calculate the healthy eating index, we estimated a proxy marker of protein from plant sources, including tofu, meat substitutes, nuts and beans. None of our participants consumed all their protein from plant sources alone; indeed 98.8% of our participants consumed ≤20% of their protein intake from plant sources (see Supplementary Figure 1 and Table 1). Thus, the results of this study should be considered in the context of a majority animal-source protein intake. This proxy measure does not include protein from other non-animal sources such as bread, though the contributions from these sources tend to be small. Indeed, the proportional contribution in our study compares with another UK study [26]. More detailed future work is needed to evaluate the impact of animal- versus plant-sourced protein on muscle health in older adults.

Only sex predicted the missingness of the protein intake variable. The literature examining sex differences in self-reported dietary intakes is mixed, with some reporting no sex differences [27, 28] and others noting differences by sex [29]; however, much of the published work in this area is focused on energy intake specifically, rather than protein intake. In our study, men had a higher proportion of missing data for protein intake than women. There is some evidence that women are more likely than men to complete questionnaires [30]. This is in keeping with our experiences within the TwinsUK cohort, particularly questionnaires that are longer and/or more laborious, such as the FFQ, and may explain some of this difference.

Renal function should be considered when advising increased protein in diet for older adults, as diets high in protein are more likely to be acidogenic in the context of age-related decline in renal function. Acidogenic diets can lead to mild metabolic acidosis, with detrimental effects on muscle mass [31], unless well balanced by plant-based alkalinogenic foods. In addition, there is some evidence that blood pH does become slightly more acidic with age [32]. The association between sarcopenia and high protein intake was robust to adjustment for both HEI, considered a proxy marker of alkalinity of diet (*P* < 0.001), and creatinine clearance (*P* = 0.034), an indicator of renal function, suggesting that diet alkalinity and renal function do not explain the association reported here. Serum creatinine and muscle mass are known to be correlated. We have used calculated creatinine clearance here, which considers weight and age, and is considered a more accurate measure of renal function, but this will still be influenced by the participant’s muscle mass. Future work examining renal function in the context of sarcopenia and dietary protein should consider other measures of renal function, such as Cystatin C [33], which are less associated with lean mass, to explore this relationship further.

In terms of BMI, those with low muscle strength had a higher BMI than those without; however, those with sarcopenia had a lower BMI than those without. The higher BMI found in the low muscle strength group may be influenced by the presence of sarcopenic obesity. This relatively new concept refers to those with muscle loss typical of sarcopenia, but with a large body mass, although a consensus definition is lacking, which makes diagnosis difficult [34]. Perhaps those with low muscle strength represent an earlier point on the pathophysiological pathway of sarcopenia development, and by the time they have reached the criteria for sarcopenia, they have lost body mass, in keeping with the typical image of a person with sarcopenia, with a thin body habitus. Further high-quality, longitudinal research is required to explore this further.

Due to a growing body of evidence linking the gut microbiota to skeletal muscle health [14], alpha diversity of the gut microbiota was included as an exposure variable, with notably less diverse gut microbiota in those with low muscle strength; however, this was not sustained for sarcopenia, perhaps due to our small number of sarcopenia cases lacking power to detect an association. When it comes to muscle health, it may well be that the function of the gut microbiota is more important than the diversity, and that diversity alone insufficiently encompasses microbiota composition and function. Ongoing trials are investigating targeting the gut microbiota to improve muscle strength [35], which will provide insights into whether the gut microbiota may represent a future therapeutic target for age-associated muscle loss and muscle strength.

Previous research in this cohort examined the heritability of muscle health and found a moderate genetic component, with heritability estimates of 0.46 for leg extensor strength, 0.3 for handgrip strength and 0.52 for lean body mass (all *P* < 0.05) [36], notably higher for mass than for strength measures. Other research investigating discordant twins for muscle strength found the stronger twins had higher physical activity [37], in keeping with the inference that muscle strength is modifiable by environment and lifestyle, rather than heavily influenced by genetics. However, the evidence linking early birth weight to later sarcopenia development [38] indicates that sarcopenia’s origins are developmental [39], highlighting the importance of twin studies in this field.

The association between muscle strength and each of the variables (weight, BMI, healthy eating index, protein intake and alpha diversity) does not appear to be significantly influenced by shared twin factors. This tentatively suggests that those variables may be more modifiable in preventing the development of sarcopenia. This is perhaps intuitive when it comes to weight and diet; however, it is promising to see that gut microbiota diversity also appears to be modifiable in this way. To our knowledge, this finding has not been shown elsewhere and can guide researchers in this field going forward, where sarcopenia research has struggled to find modifiable treatment targets.

## Strengths and limitations

Due to historical reasons, the TwinsUK cohort is predominantly female and white [15], as is the case in this study. Despite this, the cohort is largely representative of the UK population [15]; however, it does have a healthy volunteer bias. This study is cross-sectional in nature, and therefore, definitive conclusions about the direction of associations cannot be made. In addition, while the vast majority of variables were contemporaneously measured at the same visit, occasionally when no data were available for that variable, the most recent previous value was imputed. While DXA scans are a recommended and satisfactory measure of muscle mass, it is worth noting that computed tomography or magnetic resonance imaging is the gold standard [1], although whole-body measurement can be limited and costly using these methods. The low prevalence of sarcopenia, while not out of keeping with existing literature, means that the number of the individuals with sarcopenia in this study is low, reducing power, and while every effort was made to ensure the conclusions of our analyses are robust, further research with a larger number of people living with sarcopenia is warranted to investigate this further. Lastly, while chair-rise time and gait speed are also recognised and accepted by EWGSOP2 as measures of muscle strength, they are not isolated isometric muscle measures and require neurological function, adequate vision, etc., which may influence the results of these tests in some participants. The major strengths of our study are our investigation of potential factors that influence sarcopenia and exploration of shared twin influences on the factors associated with sarcopenia.

## Conclusions

We report a sarcopenia prevalence of 4.3% in a cohort of community-dwelling volunteer twins, aged ≥60 years. Key factors that influence muscle strength include age, education, income, BMI, healthy diet, physical activity, frailty, appetite, protein intake and gut microbiota diversity. The association between muscle strength and each of the variables (weight, BMI, healthy eating index, protein intake and alpha diversity) was not significantly influenced by shared twin factors. These potentially modifiable factors may therefore be more amenable to interventions aiming to prevent and/or treat sarcopenia.

High protein intake is associated with sarcopenia, even after adjustment for a range of covariates. This finding should be considered when advising increased protein intake for older adults without assessing baseline consumption. Further analysis is warranted, including longitudinal data, in cohorts with a larger number of individuals living with sarcopenia, to assess this association further.

## Supplementary Material

Appendices_supp_materials_final_afad018

## Data Availability

Data are available on request from TwinsUK, please see https://twinsuk.ac.uk/resources-for-researchers/access-our-data/. for details.
