# Supplementary material for: Higher dietary protein intake is associated with sarcopenia in older British twins
Source: Age Ageing. 2023 Feb 14;52(2):afad018. doi: 10.1093/ageing/afad018 (PMC10248216; doi:10.1093/ageing/afad018)
Supplement: Appendices_supp_materials_final_afad018 [file appendices_supp_materials_final_afad018.docx]

# **Higher dietary protein intake is associated with sarcopenia in older British twins**

# **Contents of Supplementary Materials**

1. Appendices: Methods: Variable measurement, Statistical Analysis
2. Supplementary Figure 1: Proportion of protein intake that comes from plant sources
3. Supplementary Table 1: Conversion factors used to ascertain proportion of dietary protein from plant sources
4. Supplementary Table 2: Domains included to quantify the Frailty Index
5. Supplementary Table 3: ﻿Univariable logistic regression results for covariates of low muscle strength and sarcopenia
6. Supplementary Table 4: ﻿ORs and 95% CIs for low muscle strength according to protein intake (measured as g/total lean mass). Reference category is middle tertile
7. Supplementary Table 5: Missingness of data
8. Supplementary Table 6: Multivariable logistic regression analysis for missingness of protein intake

# **Appendices**

**Methods**

### **Variable measurement**

Muscle mass was measured using DXA (Hologic Bone Densitometer QDR Horizon W, Serial Number 200884), and appendicular lean mass/height squared was calculated. Two measures of muscle strength were recorded; handgrip strength using Jamar Hydraulic Hand Dynamometer, with the best of 3 attempts recorded, using dominant hand, and chair-rise time (the time taken to rise from a chair 5 times without using hands). Gait speed (metres/second) was estimated from the time it took to walk 4 metres: with the mean of two attempts taken.

Low muscle strength (also known as probable sarcopenia) and sarcopenia were defined based on EWGSOP2 cut-off values, as was low muscle mass (Cruz-Jentoft et al., 2019a)(1)(1) . Thus, if a participant met the cut-off for reduced handgrip strength (<27 kg for males; <16 kg for females) and/or chair-rise time (>15 s for 5 chair rises), they were considered to have low muscle strength. The cut offs for muscle mass (appendicular lean mass/height squared) were <7kg/m^2^ for men and <5.5kg/m^2^ for women (Cruz-Jentoft et al., 2019a, 2019b)(1,16)(1,16). Muscle strength and mass were then used as binary categorical variables, defined as low or not low. Sarcopenia was also a binary variable, defined as sarcopenia or no sarcopenia. Activity was measured using the International Physical Activity Questionnaire (IPAQ) (Craig et al., 2003)(17)(17), which computes a score of 1-2-3 representing low-moderate-high physical activity, based on MET minutes and the volume and frequency of physical activity per week (Ipaq.ki.se, 2004)(18)(18). MET stands for metabolic equivalent of task, one MET minute is the energy expended at rest in a minute (Jetté et al., 1990)(19)(19). To determine whether appetite was associated with sarcopenia, this was measured using the Simplified Nutritional Appetite Questionnaire (SNAQ) (Wilson et al., 2005)(20)(20).

Dietary intake was measured using self-administered food frequency questionnaires (EPIC-FFQ) which was developed and validated for a UK population (Bingham et al., 2001)(21)(21), from which daily protein intake and energy intake were calculated using the validated FETA (FFQ EPIC Tool for Analysis) tool (Mulligan et al., 2014)(22)(22). The FFQ is a valid tool for estimating protein intake (Kroke et al., 1999; Okada et al., 2017)(23,24)(23,24). To understand whether diet quality impacted on associations between protein intake and sarcopenia, data from the FFQ was used to calculate the Healthy Eating Index (HEI), as described previously (Bowyer et al., 2018)(25)(25). FFQ entries were removed based on the following three criteria: (1) >10 incomplete items from the 130 food items on the FFQ as per recommendations (Mulligan et al., 2014)(22)(22) to reduce missing data error; (2) outside of 2 standard deviations (SD) (per batch, of 3) for the ratio of energy intake / basal metabolic rate (calculated using Harris-Benedict equations); (3) >2 SD of mean for macronutrients (protein, fat, and carbohydrate), both of which aim to reduce under-reporting and over-reporting. Protein was presented both as a binary variable, using the UK Reference Nutrient Intake (RNI) for adults which is ≥0.75g/kg body weight/day (“Dietary Reference Values for Food Energy and Nutrients for the United Kingdom.  Report of the Panel on Dietary Reference Values of the Committee on Medical Aspects of Food Policy.,” 1991)(26)(26), and as per the ESPEN recommended intakes for older adults, which recommends 1-1.3g/kg/day as optimal intake (Nowson et al., 2015)(12)(12), thus creating a categorical variable of low (<1g/kg body weight/day), optimal (1-1.3g/kg body weight/day) and high (>1.3g/kg body weight/day) intake. Participants were also asked to report use of any dietary supplements.

In all analyses protein intake was expressed and analysed in grams per kilogram of body weight per day, because it is most easily translated for clinicians and patients alike (who often know their body weight or can easily measure it) and also importantly because the ESPEN guidance for older adults uses this format (Nowson et al., 2015)(12)(12). In addition, in a supplementary analysis, protein intake was also expressed as grams per kilogram of total fat-free mass (FFM), as it has been suggested that this provides a more accurate representation of individual protein requirements (Dekker et al., 2022)(27)(27).

The gut microbiota was measured from one stool sample and sequenced using Illumina MiSeq as described previously (Goodrich et al., 2014)(28)(28). Alpha diversity of the gut microbiota was quantified as observed Shannon diversity index, as described previously (Jackson et al., 2016)(29)(29). Weight (kg) was measured using Marsden MPPS-250 scale, height (cm) was measured using a Leicester Height Measurer and body mass index (BMI) was calculated as weight/height squared. Frailty was quantified through the Rockwood Frailty Index (Searle et al., 2008)(30)(30), using self-reported data across 36 domains of age-related health deficits (see Supplementary Table 2). Serum creatinine was measured using a standard enzymatic rate (creatinine amidohydrolase) followed by colorimetric assay (Kodak Ektachem dry chemistry analysers, Johnson and Johnson Vitros Ektachem). Creatinine clearance (ml/min) was calculated using the Cockcroft Gault formula: 1.2 x (140 - age) x body weight[kg]/creatinine[µmol/L], with the answer multiplied by 0.85 if female (Cockcroft & Gault, 1976)(31)(31). This was included in the analysis to determine whether renal function may influence the relationship between sarcopenia and protein.

Demographic characteristics were recorded by questionnaires including smoking status, income, and education. Income referred to annual household earnings and was categorised as low (<£30,000), middle (£30,000-50,000) and high (>£50,000). Education level was categorized as low (up to GCSE or equivalent), middle (A levels, diploma) or high (university degree or higher). Available case analysis approach was used to handle missing data. For all data, the most recent collection was used and only data recorded since 2010 was included.

### **Statistical analysis**

Statistical analysis was performed using Stata (Version 15.1). Data distributions were found to be normal, which allowed the use of parametric tests. To characterise the differences between those with low/normal muscle strength, and with/without sarcopenia, continuous variables were compared with two sample t-tests and categorical variables with Pearson’s chi-squared tests. To assess for multicollinearity, correlation coefficients were checked for all variables of interest and were found to be <0.7 in all cases.

Univariable logistic regression analysis was used to determine odds ratios (ORs) for the relation between each variable and categories of low muscle strength, low muscle mass, and sarcopenia. All variables were standardised. All univariable analyses were adjusted for age and sex. Multivariable logistic regression analysis was used to determine the adjusted ORs of low muscle strength for dietary protein intake, using optimal intake (1-1.3g/kg/day) as the reference category (Nowson et al., 2015)(12)(12). Variables were selected for the multivariable model based on significance in the univariable model and/or existing evidence for an association with sarcopenia. Values of *P*<0.05 were considered statistically significant. A supplementary analysis was carried out to test whether any exposure variables of interest predicted missingness of the protein intake data, to examine whether missingness influenced the results of the logistic regression analyses.

Twins are naturally matched pairs, with shared genetics, depending on zygosity, and shared early-life experiences. This means that the data has an inherently paired structure, which induces correlation between the pairs. To adjust for this, the data in the regression models were clustered by twin pair. However, a more detailed form of analysis to investigate the relative importance of shared *versus* non-shared factors is the between-pair and within-pair model approach (Carlin et al., 2005)(32)(32). For this twin modelling analysis, the continuous variable of chair-rise time was used as a marker of muscle strength. Linear modelling was used in this analysis, considering the linear relationship between protein intake and muscle strength. The within-pair (variable_within) coefficient predicts the difference in outcome per unit difference within the pair and is free of confounding of shared twin factors. The between-pair (variable_between) coefficient predicts the difference in outcome per unit of the pair average of each predictor variable. A Wald test was used to test the difference between the between-pair and within-pair coefficients. Lone twins were excluded from this analysis.

**References**

1. Cruz-Jentoft AJ, Bahat G, Bauer J, Boirie Y, Bruyère O, Cederholm T, et al. Sarcopenia: revised European consensus on definition and diagnosis. Age Ageing. 2019;48(1):16–31.

2. Cruz-Jentoft AJ, Bahat G, Bauer J, Boirie Y, Bruyère O, Cederholm T, et al. Erratum: Sarcopenia: Revised European consensus on definition and diagnosis (Age and Ageing DOI: 10.1093/ageing/afy169) [Internet]. Vol. 48, Age and Ageing. Oxford University Press; 2019 [cited 2020 Sep 30]. p. 601. Available from: https://academic.oup.com/ageing/article/48/4/601/5488778

3. Craig CL, Marshall AL, Sjöström M, Bauman AE, Booth ML, Ainsworth BE, et al. International physical activity questionnaire: 12-Country reliability and validity. Med Sci Sports Exerc. 2003;35(8):1381–95.

4. Ipaq.ki.se. Guidelines for Data Processing and Analysis of the International Physical Activity Questionnaire (IPAQ)-Short Form [Internet]. 2004 [cited 2022 Aug 5]. Available from: www.ipaq.ki.se.

5. Jetté M, Sidney K, Blümchen G. Metabolic equivalents (METS) in exercise testing, exercise prescription, and evaluation of functional capacity. Clin Cardiol [Internet]. 1990 Aug [cited 2022 Nov 2];13(8):555–65. Available from: https://pubmed.ncbi.nlm.nih.gov/2204507/

6. Wilson MMGMG, Thomas DR, Rubenstein LZ, Chibnall JT, Anderson S, Baxi A, et al. Appetite assessment: Simple appetite questionnaire predicts weight loss in community-dwelling adults and nursing home residents. Am J Clin Nutr. 2005 Nov 1;82(5):1074–81.

7. Bingham SA, Welch AA, McTaggart A, Mulligan AA, Runswick SA, Luben R, et al. Nutritional methods in the European Prospective Investigation of Cancer in Norfolk. Public Health Nutr. 2001 Jun 2;4(3):847–58.

8. Mulligan AA, Luben RN, Bhaniani A, Parry-Smith DJ, O’Connor L, Khawaja AP, et al. A new tool for converting food frequency questionnaire data into nutrient and food group values: FETA research methods and availability. BMJ Open [Internet]. 2014 Mar [cited 2021 Aug 27];4(3):e004503. Available from: http://www.srl.cam.ac.uk/epic/epicffq/

9. Okada C, Iso H, Ishihara J, Maruyama K, Sawada N, Tsugane S. Validity and reliability of a self-administered food frequency questionnaire for the JPHC study: The assessment of amino acid intake. J Epidemiol [Internet]. 2017 May [cited 2022 Nov 2];27(5):242–7. Available from: http://linkinghub.elsevier.com/retrieve/pii/S0917504016301678

10. Kroke A, Klipstein-Grobusch K, Voss S, Möseneder J, Thielecke F, Noack R, et al. Validation of a self-administered food-frequency questionnaire administered in the European Prospective Investigation into Cancer and Nutrition (EPIC) Study: comparison of energy, protein, and macronutrient intakes estimated with the doubly labeled water,. Am J Clin Nutr [Internet]. 1999 Oct 1 [cited 2022 Oct 31];70(4):439–47. Available from: https://academic.oup.com/ajcn/article/70/4/439/4729057

11. Bowyer RCE, Jackson MA, Pallister T, Skinner J, Spector TD, Welch AA, et al. Use of dietary indices to control for diet in human gut microbiota studies. Microbiome [Internet]. 2018 Dec 25 [cited 2018 Nov 8];6(1):77. Available from: https://microbiomejournal.biomedcentral.com/articles/10.1186/s40168-018-0455-y

12. Dietary reference values for food energy and nutrients for the United Kingdom.  Report of the Panel on Dietary Reference Values of the Committee on Medical Aspects of Food Policy. Rep Health Soc Subj (Lond). 1991;41:1–210.

13. Nowson C, O’Connell S, O’Connell S. Protein requirements and recommendations for older people: A review. Nutrients [Internet]. 2015/08/20. 2015;7(8):6874–99. Available from: https://www.ncbi.nlm.nih.gov/pubmed/26287239

14. Dekker IM, van Rijssen NM, Verreijen A, Weijs PJ, de Boer WB (Elsbeth), Terpstra D, et al. Calculation of protein requirements; a comparison of calculations based on bodyweight and fat free mass. Clin Nutr ESPEN [Internet]. 2022 Apr 19 [cited 2022 Mar 18];48:378–85. Available from: https://linkinghub.elsevier.com/retrieve/pii/S2405457722000250

15. Goodrich JK, Waters JL, Poole AC, Sutter JL, Koren O, Blekhman R, et al. Human Genetics Shape the Gut Microbiome. Cell. 2014 Nov;159(4):789–99.

16. Jackson MA, Jeffery IB, Beaumont M, Bell JT, Clark AG, Ley RE, et al. Signatures of early frailty in the gut microbiota. Genome Med. 2016;8(1):8.

17. Searle SD, Mitnitski A, Gahbauer EA, Gill TM, Rockwood K. A standard procedure for creating a frailty index. BMC Geriatr. 2008;8:24.

18. Cockcroft DW, Gault MH. Prediction of creatinine clearance from serum creatinine. Nephron [Internet]. 1976 [cited 2022 Dec 22];16(1):31–41. Available from: https://pubmed.ncbi.nlm.nih.gov/1244564/

19. Carlin JB, Gurrin LC, Sterne JACA, Morley R, Dwyer T. Regression models for twin studies: A critical review. International Epidemiological Association International Journal of Epidemiology. 2005 Oct 1;34(5):1089–99.

#

**Supplementary Figure 1: Proportion of protein intake that comes from plant sources**


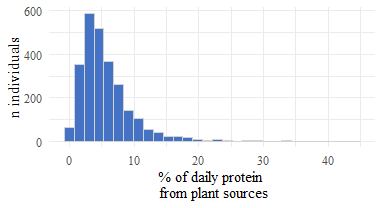


Mean 3.77 Standard deviation 2.78

*Please note this proxy measure of plant-sourced protein includes tofu, meat substitutes, nuts, and beans, and does not include all non-animal sourced protein – for example the protein in bread.

**Supplementary Table 1: Conversion factors used to ascertain proportion of dietary protein from plant sources**

| Variable Name | Description | Food Code (6th) | Food Code (5th) | Full Food Name | Proportion | Portion size | Protein (g) per portion |
| --- | --- | --- | --- | --- | --- | --- | --- |
| Tofu | Meat substitutes e.g. tofu, soyameat, textured vegetable protein, vegeburger | 13-119 | 50-723 | Tofu, soya bean, steamed | 0.4 | 120 | 9.4656 |
|  |  | 15-331 | 15-331 | Vegeburger, retail, grilled | 0.6 | 56 | 9.4656 |
| PeanutButter | Peanut butter (teaspoon) | 14-876 | 14-876 | Peanut butter, smooth | 1 | 20 | 4.56 |
| NutsSalted | Salted nuts e.g. peanuts, cashews (handful) | 14-812 | 14-812 | Cashew nuts, roasted and salted | 0.2 | 25 | 5.965 |
|  |  | 14-834 | 14-834 | Peanuts, roasted and salted | 0.8 | 25 | 5.965 |
| NutsUnsalted | Unsalted nuts, e.g. brazil, walnuts (handful) | 14-871 | 14-871 | Brazil nuts | 0.4 | 10 | 2.336 |
|  |  | 14-879 | 14-879 | Walnuts | 0.6 | 20 | 2.336 |
| Seeds | Seeds e.g. Sunflower, pumpkin (tablespoon) | 14-845 | 14-845 | Sunflower seeds | 0.5 | 16 | 3.592 |
|  |  | 14-842 | 14-842 | Pumpkin seeds | 0.5 | 16 | 3.592 |
| Peas | Peas | 13-440 | 13-440 | Peas, frozen, boiled in unsalted water | 1 | 70 | 4.2 |
| GreenBeans | Green beans, broad beans, runner beans | 13-432 | 13-432 | Green beans/French beans, frozen, boiled in unsalted water | 1 | 90 | 1.62 |
| BakedBeans | Baked beans | 13-044 | 13-044 | Baked beans, canned in tomato sauce, re-heated | 1 | 135 | 7.02 |
| Beansprouts | Beansprouts | 13-426 | 13-426 | Beansprouts, mung, raw | 1 | 20 | 0.58 |
| DriedLentils | Pulses e.g. lentils, beans, peas | 13-434 | 13-434 | Lentils, red, split, dried, boiled in unsalted water | 1 | 70 | 0.27 |

**Supplementary Table 2: Domains included to quantify the Frailty Index**

|  | Domain name | Question(s) |
| --- | --- | --- |
| 1 | **Sleep problem** | How would you describe your sleep quality over the last month? |
| 2 | **Low physical activity** | In the past year, how frequently have you typically engaged in physical exercises that raise your heart rate and last for 20 minutes at a time? (Note: You would know if an activity raised your heart rate since you would probably feel your heart beating faster, you would sweat, and/or feel out of breath) |
| 3 | **Disability** | Do you currently have a long-term disability that seriously restricts your activities? |
|  |  | Please, specify number of years you have had this disability for \ years |
|  |  | Please, specify number of years you have had this disability for \ months |
| 4 | **Dizziness** | Over the last year, have you had episodes of “dizziness” or “funny turns”? (Tick only one box) |
| 5 | **Chronic lung disease** | Has a doctor ever told you that you have/had any of the following conditions? \ Chronic bronchitis, chronic obstructive pulmonary disease (COPD) or emphysema |
|  |  | Is this an ongoing condition? \ Chronic bronchitis, chronic obstructive pulmonary disease (COPD) or emphysema |
| 6 | **Arthritis** | Has a doctor ever told you that you have/had any of the following conditions? \ Osteoarthritis (ordinary age-related arthritis) |
|  |  | Is this an ongoing condition? \ Osteoarthritis (ordinary age-related arthritis) |
|  |  | Has a doctor ever told you that you have/had any of the following conditions? \ Rheumatoid arthritis |
|  |  | Is this an ongoing condition? \ Rheumatoid arthritis |
|  |  | Has a doctor ever told you that you have/had any of the following conditions? \ Gout |
|  |  | Is this an ongoing condition? \ Gout |
|  |  | Has a doctor ever told you that you have/had any of the following conditions? \ Lupus (SLE) |
|  |  | Is this an ongoing condition? \ Lupus (SLE) |
|  |  | Has a doctor ever told you that you have/had any of the following conditions? \ Other arthritis (psoriatic arthritis, seronegative arthritis) |
|  |  | Is this an ongoing condition? \ Other arthritis (psoriatic arthritis, seronegative arthritis) |
|  |  | Has a doctor ever told you that you have/had any of the following conditions? \ Polymyalgia rheumatica |
|  |  | Is this an ongoing condition? \ Polymyalgia rheumatica |
| 7 | **Osteoporosis** | Has a doctor ever told you that you have/had any of the following conditions? \ Osteoporosis |
|  |  | Is this an ongoing condition? \ Osteoporosis |
| 8 | **Diabetes** | Has a doctor ever told you that you have/had any of the following conditions? \ Type 2 diabetes (or 'adult onset') |
| 9 | **Fragility fractures** | Have you had any of the following fractures since the age of 16? \ Hip |
|  |  | Have you had any of the following fractures since the age of 16? \ Spine |
|  |  | Have you had any of the following fractures since the age of 16? \ Wrist |
| 10 | **Falls** | How many times have you fallen in the past 6 months? A `fall’ is defined as any event that led to an unplanned, unexpected contact with a supporting surface |
| 11 | **Fatigue** | Over the past 3 months, have you often felt tired or fatigued? |
|  |  | Does tiredness or fatigue significantly limit your activities? |
| 12 | **Cardiac disease** | Has a doctor ever told you that you have/had any of the following conditions? \ Congestive heart failure |
|  |  | Is this an ongoing condition? \ Congestive heart failure |
|  |  | Has a doctor ever told you that you have/had any of the following conditions? \ Angina |
|  |  | Is this an ongoing condition? \ Angina |
|  |  | Has a doctor ever told you that you have/had any of the following conditions? \ Atrial fibrillation |
|  |  | Is this an ongoing condition? \ Atrial fibrillation |
|  |  | Has a doctor ever told you that you have/had any of the following conditions? \ Coronary heart disease |
|  |  | Is this an ongoing condition? \ Coronary heart disease |
|  |  | Has a doctor ever told you that you have/had any of the following conditions? \ Congenital heart disease (Heart valve problems) |
|  |  | Is this an ongoing condition? \ Congenital heart disease (Heart valve problems) |
| 13 | **Cardiac risk factors** | Has a doctor ever told you that you have/had any of the following conditions? \ Hypertension (high blood pressure) |
|  |  | Is this an ongoing condition? \ Hypertension (high blood pressure) |
|  |  | Has a doctor ever told you that you have/had any of the following conditions? \ High cholesterol |
|  |  | Is this an ongoing condition? \ High cholesterol |
|  |  | Has a doctor ever told you that you have/had any of the following conditions? \ A heart murmur |
|  |  | Is this an ongoing condition? \ A heart murmur |
|  |  | Has a doctor ever told you that you have/had any of the following conditions? \ A heart attack (Myocardial infarction) |
| 14 | **Venous disease** | Has a doctor ever told you that you have/had any of the following conditions? \ Deep vein thrombosis (DVT) |
|  |  | Is this an ongoing condition? \ Deep vein thrombosis (DVT) |
|  |  | Has a doctor ever told you that you have/had any of the following conditions? \ Varicose veins |
|  |  | Is this an ongoing condition? \ Varicose veins |
|  |  | Has a doctor ever told you that you have/had any of the following conditions? \ Pulmonary embolism (PE) |
|  |  | Is this an ongoing condition? \ Pulmonary embolism (PE) |
| 15 | **Gastrointestinal disease** | Has a doctor ever told you that you have/had any of the following conditions? \ Stomach or Duodenal ulcer (diagnosed with an Endoscopy or Barium Test) |
|  |  | Is this an ongoing condition? \ Stomach or Duodenal ulcer (diagnosed with an Endoscopy or Barium Test) |
|  |  | Has a doctor ever told you that you have/had any of the following conditions? \ Polyps in the colon or rectum |
|  |  | Is this an ongoing condition? \ Polyps in the colon or rectum |
|  |  | Has a doctor ever told you that you have/had any of the following conditions? \ Diverticular disease |
|  |  | Is this an ongoing condition? \ Diverticular disease |
|  |  | Has a doctor ever told you that you have/had any of the following conditions? \ Gallstones/cholelithiasis |
|  |  | Is this an ongoing condition? \ Gallstones/cholelithiasis |
| 16 | **Cancer** | What kind(s) of cancer have you been diagnosed with? \ Bladder |
|  |  | Is this an ongoing condition? \ Bladder |
|  |  | What kind(s) of cancer have you been diagnosed with? \ Brain |
|  |  | Is this an ongoing condition? \ Brain |
|  |  | What kind(s) of cancer have you been diagnosed with? \ Breast |
|  |  | Is this an ongoing condition? \ Breast |
|  |  | What kind(s) of cancer have you been diagnosed with? \ Cervix |
|  |  | Is this an ongoing condition? \ Cervix |
|  |  | What kind(s) of cancer have you been diagnosed with? \ Colon |
|  |  | Is this an ongoing condition? \ Colon |
|  |  | What kind(s) of cancer have you been diagnosed with? \ Kidney |
|  |  | Is this an ongoing condition? \ Kidney |
|  |  | What kind(s) of cancer have you been diagnosed with? \ Leukaemia |
|  |  | Is this an ongoing condition? \ Leukaemia |
|  |  | What kind(s) of cancer have you been diagnosed with? \ Lung |
|  |  | Is this an ongoing condition? \ Lung |
|  |  | What kind(s) of cancer have you been diagnosed with? \ Lymphoma |
|  |  | Is this an ongoing condition? \ Lymphoma |
|  |  | What kind(s) of cancer have you been diagnosed with? \ Oesophagus |
|  |  | Is this an ongoing condition? \ Oesophagus |
|  |  | What kind(s) of cancer have you been diagnosed with? \ Ovary |
|  |  | Is this an ongoing condition? \ Ovary |
|  |  | What kind(s) of cancer have you been diagnosed with? \ Prostate |
|  |  | Is this an ongoing condition? \ Prostate |
|  |  | What kind(s) of cancer have you been diagnosed with? \ Skin (non-melanoma) |
|  |  | Is this an ongoing condition? \ Skin (non-melanoma) |
|  |  | What kind(s) of cancer have you been diagnosed with? \ Skin (melanoma) |
|  |  | Is this an ongoing condition? \ Skin (melanoma) |
|  |  | What kind(s) of cancer have you been diagnosed with? \ Uterus |
|  |  | Is this an ongoing condition? \ Uterus |
|  |  | What kind(s) of cancer have you been diagnosed with? \ Other kind of cancer |
|  |  | Is this an ongoing condition? \ Other kind of cancer |
| 17 | **Incontinence** | Have you EVER regularly leaked urine, also known as suffering from incontinence? |
|  |  | Do you currently leak urine regularly? |
| 18 | **Neurological disease** | Has a doctor ever told you that you have/had any of the following conditions? \ Bipolar disorder (manic depression) |
|  |  | Is this an ongoing condition? \ Bipolar disorder (manic depression) |
|  |  | Has a doctor ever told you that you have/had any of the following conditions? \ Anxiety or stress disorder |
|  |  | Is this an ongoing condition? \ Anxiety or stress disorder |
|  |  | Has a doctor ever told you that you have/had any of the following conditions? \ Clinical depression |
|  |  | Is this an ongoing condition? \ Clinical depression |
|  |  | Has a doctor ever told you that you have/had any of the following conditions? \ Stroke or Transient ischemic attack (TIA) |
|  |  | Is this an ongoing condition? \ Stroke or Transient ischemic attack (TIA) |
|  |  | Has a doctor ever told you that you have/had any of the following conditions? \ Parkinson’s disease |
| 19 | **Subjective memory impairment** | Has a doctor ever told you that you have/had any of the following conditions? \ Alzheimer’s disease |
|  |  | Has a doctor ever told you that you have/had any of the following conditions? \ Memory loss |
|  |  | Is this an ongoing condition? \ Memory loss |
|  |  | During the past 12 months, have you experienced confusion or memory loss that is happening more often or is getting worse? |
|  |  | To what extent does memory loss currently affect your day-to-day life? |
| 20 | **Eye disease** | Has a doctor or an optician ever told you that you have/had any of the following conditions? \ Glaucoma |
|  |  | Is this an ongoing condition? \ Glaucoma |
|  |  | Has a doctor or an optician ever told you that you have/had any of the following conditions? \ Age-related macular degeneration (AMD) |
|  |  | Has a doctor or an optician ever told you that you have/had any of the following conditions? \ Cataract |
| 21 | **Glasses** | Do you wear spectacles/contact lenses? |
| 22 | **Hearing loss** | Do you suffer from hearing loss? |
| 23 | **Thyroid disease** | Has a doctor ever told you that you have/had any of the following conditions? \ Hyperthyroidism (overactive thyroid, characterized by weight loss) |
|  |  | Is this an ongoing condition? \ Hyperthyroidism (overactive thyroid, characterized by weight loss) |
|  |  | Has a doctor ever told you that you have/had any of the following conditions? \ Hypothyroidism (underactive thyroid, characterized by weight gain) |
|  |  | Is this an ongoing condition? \ Hypothyroidism (underactive thyroid, characterized by weight gain) |
| 24 | **Overweight** | What is your current height? (Only one measurement type is required) \ feet |
|  |  | What is your current height? (Only one measurement type is required) \ inches |
|  |  | What is your current height? (Only one measurement type is required) \ centimetres |
|  |  | What is your current height? (Only one measurement type is required) \ Don't know |
|  |  | What is your current weight? (Only one measurement type is required) \ stones |
|  |  | What is your current weight? (Only one measurement type is required) \ pounds |
|  |  | What is your current weight? (Only one measurement type is required) \ kilograms |
|  |  | What is your current weight? (Only one measurement type is required) \ Don't know |
|  |  | Is this an ongoing condition? \ Cataract |
| 25 | **Weight loss** | Over the past 6 months have you LOST more than 10 lbs (4 kg) in weight without trying to? |
| 26 | **Poor General health** | In general, would you say your health is: excellent \ very good \ good \ fair \ poor |
| 27 | **Physical function limitation** | The following items are about activities you might do during a typical day. Does your health now limit you in these activities? If so, how much? \ Moderate activities, such as moving a table, pushing a vacuum cleaner, bowling, or playing golf |
| 28 | **ADL limitation** | The following items are about activities you might do during a typical day. Does your health now limit you in these activities? If so, how much? \ Climbing several flights of stairs |
| 29 | **Occupational limitation** | During the past 4 weeks, have you had any of the following problems with your work or other regular daily activities as a result of your physical health? \ Accomplished less than you would like |
|  |  | During the past 4 weeks, have you had any of the following problems with your work or other regular daily activities as a result of your physical health? \ Were limited in the kind of work or other activities |
| 30 | **Emotional limitation** | During the past 4 weeks, have you had any of the following problems with your work or other regular daily activities as a result of any emotional problems (such as feeling depressed or anxious)? \ Accomplished less than you would like |
|  |  | During the past 4 weeks, have you had any of the following problems with your work or other regular daily activities as a result of any emotional problems (such as feeling depressed or anxious)? \ Didn’t do work or other activities as carefully as usual |
| 31 | **Pain** | During the past 4 weeks, how much did pain interfere with your normal work (including both work outside the home and housework)? |
|  |  | In the past 3 months, have you had pain in your muscles, bones, or joints lasting at least 1 week? |
|  |  | Has this pain actually lasted more than 3 months? |
| 32 | **Mental health problem** | These questions are about how you feel and how things have been with you during the past 4 weeks. For each question, please give the one answer that comes closest to the way you have been feeling. How much of the time during the past 4 weeks… \ Have you felt calm and peaceful? |
| 33 | **Low Energy** | These questions are about how you feel and how things have been with you during the past 4 weeks. For each question, please give the one answer that comes closest to the way you have been feeling. How much of the time during the past 4 weeks… \ Did you have a lot of energy? |
| 34 | **Mood disorder** | These questions are about how you feel and how things have been with you during the past 4 weeks. For each question, please give the one answer that comes closest to the way you have been feeling. How much of the time during the past 4 weeks… \ Have you felt downhearted and blue? |
| 35 | **Social activity limitation** | During the past 4 weeks, how much of the time has your physical health or emotional problems interfered with your social activities (like visiting friends, relatives, etc.)? |
| 36 | **Polypharmacy** | Names of your currently prescribed medication(s) including hormone treatments (1). 'Currently prescribed' means medications/supplements/hormones that are currently taken on an intermittent or continued basis. |

**Supplementary Table 3: ﻿Univariable logistic regression results for covariates of low muscle strength and sarcopenia**

|  | Low Muscle Strength | | Sarcopenia |  |
| --- | --- | --- | --- | --- |
| Variable | **OR (95% CI)** | **p value** | **OR (95% CI)** | **p value** |
| Age | 2.20 (1.94-2.49) | *P<0.001* | 2.41 (1.96-2.98) | P<0.001 |
| Sex | 0.97 (0.86-1.10) | P=0.624 | 1.17 (0.97-1.40) | P=0.096 |
| Smoking | 1.01 (0.90-1.14) | P=0.812 | 1.13 (0.93-1.38) | P=0.218 |
| Income | 0.83 (0.73-0.94) | *P=0.004* | 0.92 (0.73-1.16) | P=0.465 |
| Education | 0.78 (0.68-0.89) | *P<0.001* | 0.79 (0.63-1.00) | *P=0.047* |
| Height | 0.63 (0.54-0.74) | *P<0.001* | 0.63 (0.50-0.80) | *P<0.001* |
| Weight | 1.23 (1.09-1.38) | *P=0.001* | 0.33 (0.26-0.41) | *P<0.001* |
| BMI | 1.39 (1.25-1.54) | *P<0.001* | 0.45 (0.36-0.55) | *P<0.001* |
| Serum creatinine | 0.95 (0.83-1.08) | P=0.451 | 0.62 (0.47-0.81) | *P<0.001* |
| Frailty index | 1.92 (1.70-2.15) | *P<0.001* | 1.10 (0.92-1.33) | P=0.286 |
| Muscle mass | 1.02 (0.86-1.21) | P=0.797 | 0.70 (0.57-0.87) | *P=0.001* |
| Gait speed | 0.41 (0.36-0.47) | *P<0.001* | 0.92 (0.69-1.21) | P=0.541 |
| Physical activity (IPAQ) | 0.83 (0.70-0.99) | *P=0.036* | 0.94 (0.76-1.17) | P=0.590 |
| Health eating index | 0.87 (0.76-0.99) | *P=0.037* | 1.39 (1.15-1.70) | *P=0.001* |
| Protein/body weight (g/kg/day) | 0.86 (0.74-1.01) | P=0.059 | 0.83 (0.66-1.04) | P=0.106 |
| Protein/total lean mass (g/kg/day) | 0.97 (0.75-1.26) | P=0.828 | 2.04 (1.45-2.88) | *P<0.001* |
| Energy intake (kcal/day) | 0.87 (0.76-1.01) | P=0.062 | 0.79 (0.62-0.99) | *P=0.045* |
| Appetite (SNAQ) | 0.62 (0.53-0.73) | *P<0.001* | 0.96 (0.78-1.20) | P=0.735 |
| Alpha diversity (Shannon) | 0.80 (0.70-0.92) | *P=0.001* | OR (95% CI) | p value |

BMI: Body Mass Index; IPAQ: International Physical Activity Questionnaire; SNAQ: Simplified Nutritional Appetite Questionnaire. All results are adjusted for age and sex.

**Supplementary Table 4: ﻿ORs and 95% CIs for low muscle strength according to protein intake (measured as g/total lean mass). Reference category is middle tertile**

|  | Low Muscle Strength | | Sarcopenia |  |
| --- | --- | --- | --- | --- |
| Protein/total lean mass (g/kg/day) | **Low tertile** | **High tertile** | **Low tertile** | **High tertile** |
| Unadjusted | 0.82 (0.57-1.16) | 1.04 (0.74-1.47) | 0.59 (0.30-1.15) | 1.91 (1.14-3.20) |
|  | P=0.261 | P=0.828 | P=0.121 | *P=0.014* |
| Model 1  *age, sex* | 0.93 (0.64-1.36) | 0.99 (0.69-1.42) | 0.55 (0.26-1.16) | 2.17 (1.27-3.71) |
|  | P=0.708 | P=0.954 | P=0.116 | *P=0.005* |
| Model 2  *1 + smoking, income, education* | 0.86 (0.57-1.29) | 1.00 (0.68-1.47) | 0.48 (0.21-1.12) | 2.37 (1.35-4.17) |
|  | P=0.459 | P=0.993 | P=0.089 | *P=0.003* |
| Model 3  *2 + height* | 0.89 (0.59-1.34) | 0.87 (0.59-1.28) | 0.50 (0.21-1.15) | 2.20 (1.26-3.85) |
|  | P=0.572 | P=0.480 | P=0.104 | *P=0.006* |
| Model 4: frailty/activity  *2 + frailty index + activity level (IPAQ)* | 1.01 (0.60-1.69) | 1.04 (0.63-1.74) | 0.40 (0.13-1.25) | 2.15 (1.01-4.57) |
|  | P=0.965 | P=0.869 | P=0.115 | *P=0.047* |
| Model 5: muscle  *4 + lean mass/height2* | 0.93 (0.54-1.58) | 1.06 (0.63-1.79) | 0.44 (0.14-1.38) | 1.26 (0.53-2.99) |
|  | P=0.777 | P=0.836 | P=0.161 | P=0.593 |
| Model 6: renal function  *4 + serum creatinine* | 0.98 (0.58 – 1.65) | 1.01 (0.60-1.69) | 0.42 (0.13-1.36) | 2.05 (0.97-4.37) |
|  | P=0.935 | P=0.977 | P=0.149 | P=0.061 |
| Model 7: diet  *2 + energy intake (kcal/day), healthy eating index* | 0.69 (0.43-1.09) | 1.21 (0.71-1.91) | 0.22 (0.08-0.55) | 4.53 (2.29-8.96) |
|  | P=0.111 | P=0.424 | *P=0.001* | *P<0.001* |
| Model 8: diet  *6 + SNAQ score* | 0.61 (0.32-1.17) | 1.51 (0.82-2.77) | 0.12 (0.03-0.47) | 5.22 (2.08-13.09) |
|  | P= 0.137 | P=0.184 | *P=0.003* | *P<0.0001* |
| Model 9: Muscle & Gut Microbiome  4 + shannon diversity | 1.29 (0.69-2.43) | 1.50 (0.80-2.79) | 0.58 (0.14-2.47) | 3.86 (1.34-11.07) |
|  | P=0.422 | P=0.206 | P=0.465 | *P=0.012* |

IPAQ: International Physical Activity Questionnaire; SNAQ: Simplified Nutritional Appetite Questionnaire.

**Supplementary Table 5: Missingness of data**

| Variable | Missing | Total | Percent Missing |
| --- | --- | --- | --- |
| Age | 0 | 3,302 | 0 |
| Sex | 0 | 3,302 | 0 |
| Muscle strength | 0 | 3,302 | 0 |
| Height | 1 | 3,302 | 0.03 |
| Weight | 4 | 3,302 | 0.12 |
| Body Mass Index (BMI) | 5 | 3,302 | 0.15 |
| Smoking status | 36 | 3,302 | 1.09 |
| Serum creatinine | 55 | 3,302 | 1.67 |
| Muscle mass (appendicular lean mass/height^2^) | 124 | 3,302 | 3.76 |
| Frailty index | 261 | 3,302 | 7.9 |
| Education | 307 | 3,302 | 9.3 |
| Gait speed | 340 | 3,302 | 10.3 |
| Income | 442 | 3,302 | 13.39 |
| Protein intake (g/d) | 705 | 3,302 | 21.35 |
| Energy intake (kcal/d) | 705 | 3,302 | 21.35 |
| Shannon diversity of the gut microbiome | 1,306 | 3,302 | 39.55 |
| Appetite (SNAQ score) | 1,510 | 3,302 | 45.73 |
| Physical activity (IPAQ score) | 1,909 | 3,302 | 57.81 |

SNAQ: Simplified Nutritional Assessment Questionnaire. IPAQ: International Physical Activity Questionnaire.

**Supplementary Table 6: Multivariable logistic regression analysis for missingness of protein intake**

| Variable | OR (95% CI) | p value |
| --- | --- | --- |
| Age | 1.19 (0.75-1.89) | P=0.452 |
| Sex | 1.86 (1.06-3.24) | P=0.029 |
| Zygosity | 1.07 (0.54-2.14) | P=0.840 |
| Income | 0.99 (0.69-1.43) | P=0.961 |
| Education | 0.71 (0.49-1.03) | P=0.069 |
| Height | 0.86 (0.05-14.07) | P=0.914 |
| Weight | 0.72 (0.001-304.96) | P=0.914 |
| BMI | 2.04 (0.01-487.41) | P=0.799 |
| Serum creatinine | 1.22 (0.84-1.78) | P=0.305 |
| Frailty index | 1.36 (0.86-2.14) | P=0.183 |
| Muscle mass | 0.62 (0.28-1.41) | P=0.256 |
| Gait speed | 1.10 (0.70-1.72) | P=0.676 |
| Chair rise time | 1.01 (0.59-1.73) | P=0.966 |
| Sarcopenia | 0.23 (0.02-2.43) | P=0.220 |
| Physical activity (IPAQ) | 1.35 (0.94-1.95) | P=0.103 |
| Health eating index | 0.75 (0.52-1.10) | P=0.141 |
| Appetite (SNAQ) | 1.10 (0.72-1.67) | P=0.674 |
| Alpha Diversity (shannon) | 0.88 (0.61-1.27) | P=0.493 |

BMI: Body Mass Index; IPAQ: International Physical Activity Questionnaire; SNAQ: Simplified Nutritional Appetite Questionnaire
